# Public and professional stakeholders’ perceptions of alcohol advertising and availability policies: A qualitative study

**DOI:** 10.1111/dar.13972

**Published:** 2024-10-28

**Authors:** Elena D. Dimova, Niamh K. Shortt, Matt Smith, Richard J. Mitchell, Peter Lekkas, Jamie R. Pearce, Tom L. Clemens, Carol Emslie

**Affiliations:** ^1^ Glasgow Caledonian University Glasgow UK; ^2^ Centre for Research on Environment, Society and Health, School of GeoSciences, University of Edinburgh Edinburgh UK; ^3^ MRC/CSO Social and Public Health Sciences Unit Institute for Health and Wellbeing, University of Glasgow Glasgow UK

**Keywords:** advertising, alcohol, availability, policy, public health

## Abstract

**Introduction:**

Reducing alcohol availability and restricting alcohol advertising are effective ways to reduce harm from alcohol. Implementation of public health policies involves collaboration between different stakeholders, and is influenced by public opinion. This paper explores public and professional stakeholders' perceptions of alcohol advertising and availability policies. It is the first to capture consensus and divergence in narratives of these stakeholders.

**Methods:**

We conducted semi‐structured interviews with 14 stakeholders from third sector organisations, government, public health and alcohol licensing in Scotland. We conducted 11 online focus groups with 45 participants, living in neighbourhoods in Scotland characterised by varying levels of urbanity, deprivation and retail density change. We gave participants a list of policies and discussed their views on acceptability, feasibility and likely success.

**Results:**

Despite general consensus that regulation of alcohol advertising is an important priority, public stakeholders were concerned about the feasibility of advertising interventions and potential unintended consequences. While professional stakeholders were in favour of regulating alcohol availability, public stakeholders had misgivings about feasibility and effectiveness. When prompted to discuss specific interventions, similar views about protecting children and achieving cultural change emerged.

**Discussion and Conclusions:**

This study highlights the importance of policy makers and other stakeholders to consider public stakeholders' opinions on alcohol policy and understanding that their views may be influenced by competing framings of alcohol problems. Attempts to increase support for alcohol control policies need to consider people's concerns about the effectiveness and potential unintended consequences of these policies, and the wider social context of alcohol consumption.

## 1 INTRODUCTION

Alcohol is a leading risk factor for disease burden and preventable mortality worldwide [1]. Population‐level policies to address alcohol consumption have the potential to reduce premature mortality and morbidity, and narrow health inequalities [2]. The World Health Organization [3, 4] recommends reducing alcohol advertising, availability and affordability as evidence‐based, cost‐effective ways to prevent alcohol‐attributable diseases. Policies to regulate alcohol advertising include restrictions on content, frequency and placement [4]. Interventions addressing alcohol availability include spatial (e.g., exclusion zones around schools; reduced number of retail outlets) and temporal (e.g., reducing hours of sales) restrictions [2, 5]. Pricing policies include setting minimum prices and increasing taxes on alcohol [6, 7].

Implementation of public health policies involves collaboration between policy makers and other stakeholders (e.g., advocacy groups, third sector organisations), and is influenced by public opinion [8], with policy makers more likely to pursue policies that are widely accepted in society [9]. Alcohol control policies can be highly contested [10, 11, 12] and negative public attitudes may lead to governments withdrawing support for policies (e.g., [13]). Alcohol industry actors are often active in shaping the public discourse about alcohol and its effects on society [14]. Motivated by increasing their profits, they tend to focus on ‘responsible drinking’ and frame alcohol problems in narrow individualised terms, suggesting only a minority of the population experiences harm from alcohol [15]. In contrast, non‐industry actors (e.g., non‐government organisations and advocacy groups) characterise alcohol as a policy issue that negatively affects the whole society [15]. Previous research suggests that the most effective population‐level alcohol policies (e.g., addressing price and availability of alcohol) have proven less popular among the public, compared to lighter touch policies (e.g., mass media campaigns), which are generally less effective [16, 17, 18]. In addition, public health guidance often relies on quantitative evidence generated from epidemiological studies and fails to consider lay knowledge and perspectives, as well as subjective experience, which can limit the effectiveness of such guidance [19, 20]. For example, when drinking guidelines are framed solely within epidemiological paradigms (e.g., recommended daily unit amount), they do not always consider the context in which people drink or the value people attribute to drinking, such as pleasure. Therefore, guidelines can be viewed as lacking relevance to everyday life and are often disregarded by adult drinkers, who monitor their drinking according to their own knowledge and experience [20].

Lay perspectives are critical in ensuring effective alcohol‐related policy making. They provide important knowledge about the ‘meaning’ of alcohol behaviours in everyday contexts [20, 21], people's interpretation of scientific knowledge [22] and its relevance to everyday practices of drinking [23]. They also provide understanding about how members of the public view the role of government and other actors in alcohol control policies [24]. As Popay and Williams [25] highlight, lay people have an “‘expert’ body of knowledge, different from but equal to that of professionals in the public health field” (p. 760), and their insight can enhance the credibility and efficacy of public health interventions. Policy acceptability is shaped by people's knowledge and perceived credibility of evidence, the social context in which the policy is to be implemented and who is likely to be affected. For example, Li et al. [26] found that attitudes towards alcohol control policies are influenced by people's own identity (e.g. if they saw themselves as responsible drinkers) and how they view others around them. Lonsdale et al. [13] attributed public dislike for minimum unit pricing to misunderstanding of the policy and preoccupation with its effect on heavy and dependent drinkers. They suggest that addressing inaccurate beliefs and explaining the aim of reducing alcohol‐related harm across the whole population could help to improve the acceptance of population‐level policies among the public [13]. However, focusing on policies which align with public perceptions may lead to a neglect of evidence‐based approaches [20]. On the other hand, lack of public support for alcohol policy may result in unintended, negative consequences and failure of the policy to produce desired outcomes [27]. Given the salience of alcohol in Western societies and the dynamic relationship between public opinion and policy [28], it is important to understand how public health stakeholders and the public view alcohol control policies. Understanding public opinions on alcohol policy can inform the way policy makers and public health actors engage with the public to gain support for policy implementation.

The focus of this study is Scotland where 22% of the population consume alcohol at hazardous or harmful levels, and alcohol‐related harm is significantly higher than other countries of the United Kingdom [29]. In 2022, there were 1276 alcohol‐specific deaths and 35,187 alcohol‐related hospital admissions with rates six times higher for people living in the most deprived neighbourhoods compared with the least deprived [30]. This paper explores public and professional stakeholders’ attitudes towards potential policy interventions in two of the three areas recommended by the World Health Organization: restrictions on alcohol advertising and regulating alcohol availability. We presented participants with a list of specific policy interventions and discussed their views on acceptability, feasibility and likely success. We did not focus on the third World Health Organization recommendation, alcohol affordability, as minimum unit pricing was implemented in Scotland in 2018 and has been substantially evaluated [31]. Public health actors played a key role in the advocacy and implementation of minimum unit pricing [32]. Minimum unit pricing has been extensively evaluated in Scotland [33] and studies have included professional (e.g., [34]), industry (e.g., [35]) and public stakeholders’ views (e.g., [36, 37]). This paper makes a unique contribution to existing literature, as it is the first to explore consensus and divergence in narratives of public and professional stakeholders.

## 2 METHODS

The analysis is based on data from semi‐structured interviews with professional stakeholders and focus groups with community members living in different neighbourhoods across Scotland. The present research is part of a mixed‐methods study examining the potential link between alcohol and tobacco availability and consumption [34, 38, 39]. Ethical approval was granted by the Nursing Department Research Ethics Committee at Glasgow Caledonian University (HLS/NCH/20/033, June 2021; amendment in November 2022).

### 2.1 Sample and recruitment

#### 2.1.1 Professional stakeholders

We used pragmatic purposive sampling and snowball referrals [40] to recruit professional stakeholders who had interest and experience in alcohol policy, and were familiar with the policy landscape in Scotland (for more details, see [38]). Potential participants were identified through professional networks, then emailed and invited to take part. The respondents (*n* = 14, 10 women, 4 men) were from third sector, policy, public health and alcohol licensing. All the professionals either had policy influence (e.g., informing policy development, implementing policies made by others) and/or were involved in advocacy with frequent communication with policymakers. Time in current role varied from 4 months to 17 years. Three of these respondents were involved in the development of policy suggestions, discussed in this paper.

We also invited retailers (supermarkets and convenience stores) to take part in the study but all declined or did not respond. Those who declined to participated reported that mitigating the impact of the COVID‐19 pandemic was taking priority.

#### 2.1.2 Public stakeholders

The public stakeholders included members of the public, living in different neighbourhoods in Scotland. The sampling frame included more and less socio‐economically deprived communities (given differential impacts of retail environment within them, [41]), change in alcohol outlet availability over time and rural/urban classification. We gathered data on the location of outlets licensed to sell alcohol at two time points, 2012 and 2020. The 2012 data was obtained from local liquor licensing boards. The 2020 data was retrieved from Police Scotland's Inn Keepers Database. Data for both years included the individual postcode of each outlet allowing us to georeference their locations and allocated outlets to datazones.^i^ For each data zone we also captured: urban/rural status (binary, based on the Scottish Government's official classification); socio‐economic deprivation level (quintile, based on the Scottish Index of Deprivation Income domain, which reflects the proportion of the resident population in receipt of means tested benefits); and the absolute change in retail density for alcohol on sales, alcohol off sales outlets between 2012 and 2020. Density was measured using a kernel density estimation approach, following previous studies [42, 43]. Kernel density estimation converts a set of retailer locations into a continuous surface that considers both the number of retailers and the distance between them. We used a 50 m × 50 m grid and an 800 m kernel search radius. We linked the kernel density estimation surface for 2012 and 2020 to the datazones and computed the absolute change.

We then worked through the sampling frame to determine first, whether specific combinations of urbanity, deprivation and retail density change (either an increase or decrease in availability) in the sampling frame existed in Scotland, and then whether we could identify places with clusters of adjacent datazones fulfilling those criteria. There were some combinations of urbanity, deprivation and change in density that were not identified. For example, there were no urban deprived locations with substantial falls in both alcohol on and off retail density. Where more than one suitable cluster of datazones was identified, we considered accessibility and efficiency of fieldwork as secondary criteria to assist our selection. This process created sets of datazones with the various combinations of urbanity, deprivation and retail density change. Finally, we identified and extracted all residential postcodes that lay within these sets of datazones. We worked with two marketing agencies (Acumen and Roots) to recruit participants residing in the pre‐specified postcode areas, ensuring a mixture of age, gender and health behaviours (i.e., alcohol use and smoking) (see Table 1 for participants’ characteristics). Overall, 28 women and 17 men took part. Ages ranged from 22 to 70 years, with mean age of 45 years.

**TABLE 1:** Participant characteristics.

Focus group (no. of participants)	Age, range	Type of group^a^	Gender	AUDIT‐C scores
FG1 (=3*) *1 individual interview	37–42 years	Rural, deprived, increased tobacco retail density	Mixed (2 female, 1 male)	Low risk (*n* = 2) Higher risk (*n* = 1)
FG2 (*n* = 2)	57, 67 years	Rural, affluent, decreased alcohol on‐premise and off‐premise retail density	Men	Low risk (*n* = 2)
FG3 (*n* = 6)	30–65+ years	Urban, deprived, increased alcohol on‐premise and off‐premise retail density	Mixed (5 female, 1 male)	Non‐drinker (*n* = 1) Low risk (*n* = 5)
FG4 (*n* = 7)	24–55 years	Urban, affluent, increased alcohol on‐premise and off‐premise retail density	Mixed (5 female, 2 male)	Non‐drinker (*n* = 1) Low risk (*n* = 1) Increasing risk (*n* = 5)
FG5 (*n* = 2)	27, 45 years	Urban, affluent, alcohol on‐premise up, alcohol off‐premise up	Mixed (1 female, 1 male)	Increasing risk (*n* = 1) Higher risk (*n* = 1)
FG6 (*n* = 6)	26–69 years	Urban, deprived, increased tobacco retail density	Mixed (4 female, 2 male)	Non‐drinker (*n* = 1) Low risk (*n* = 1) Increasing risk (*n* = 4)
FG7 (*n* = 4)	38–52 years	Urban, deprived, decreased tobacco retail density	Mixed (3 female, 1 male)	Higher risk (*n* = 4)
FG8 (*n* = 5)	29–63 years	Urban, affluent, decreased tobacco retail density	Mixed (2 female, 3 male)	Non‐drinker (*n* = 1) Low risk (*n* = 2) Increasing risk (*n* = 2)
FG9 (*n* = 3)	22–61 years	Urban, affluent, increased tobacco retail density	Mixed (2 female, 1 male)	Low risk (*n* = 1) Increasing risk (*n* = 1) Higher risk (*n* = 1)
FG10 (*n* = 4*) *1 individual interview	58–70 years	Rural, deprived, decreased tobacco retail density	Mixed (2 female, 2 male)	Increasing risk (*n* = 1) Higher risk (*n* = 3)
FG11 (*n* = 3)	30–42 years	Urban, deprived, increased alcohol on‐premise and off‐premise retail density	Mixed (2 female, 1 male)	Higher risk (*n* = 3)

Abbreviation: AUDIT‐C, Alcohol Use Disorders Identification Test.

^a^ The focus groups belong to specific data zones, chosen based on urban/rural status; socio‐economic deprivation level (SIMD quintiles 1 and 2, classified as ‘deprived’; 4 and 5 as ‘affluent’); the absolute change in retail density for alcohol on sales, alcohol off sales outlets, and tobacco outlets, between 2012 and 2020 (based on the count of retailers per 1000 population).

### 2.2 Data collection

The interview and focus groups schedules explored participants’ views on potential policies to address alcohol advertising and availability in Scotland. Participants were asked to discuss each policy suggestion in terms of its perceived feasibility and likely success in addressing alcohol use and related harms.

Initial policy ideas were developed based on a scoping exercise of existing literature [11, 44, 45, 46]. These were refined through consultation and iterative review with third sector and public health stakeholders, and the research team. First, a version of the suggestions was sent to the stakeholders and a meeting held to discuss their views. Then the suggestions were discussed by the research team and finalised. We worked with a design company to produce infographics, which were used during the interviews and focus groups (Supporting information 1). The policy suggestions, discussed in this study, were:

1. Restrict alcohol advertising and sponsorship of sporting events and festivals, and restrict alcohol advertising in public spaces such as streets, parks and on public transport.
2. Ban the sale of alcohol near schools and playparks.
3. Limit the number of places which sell alcohol in neighbourhoods where there are already lots of places to buy it (i.e. areas of high density).
4. Alcohol should only be sold in one area of the shop which is hidden by a physical barrier.

#### 2.2.1 Professional stakeholders

We conducted 11 interviews with 14 participants (10 one‐to‐one, 1 with four stakeholders from the same organisation) between October 2021 and February 2022. Interviews were considered appropriate as professional stakeholders’ views are grounded in their unique experience within specific organisations and involvement in the policy context. Due to the COVID‐19 pandemic, all interviews were conducted remotely via a video call. Interview duration varied from 40 to 82 min, with an average length of 57 min. A study information leaflet and a consent form were given to participants and informed consent recorded prior to the interview. To maintain anonymity, each respondent was assigned a unique ID number.

#### 2.2.2 Public stakeholders

We conducted 11 online focus groups with 45 participants between August 2022 and May 2023. Focus groups were chosen in order to explore the appetite for specific policy suggestions from the perspectives of people living in the same neighbourhood. Previous research shows that perceptions of policies can change during group discussions and after contextualising a policy within a specific social setting [26]. Focus group duration varied from 66 to 115 min, with an average length of 92 min. Each participant was reimbursed with a £40 Love2Shop voucher. Transcripts were anonymised and participants given pseudonyms.

In the findings section of this paper, we sometimes refer to the public stakeholders as “focus group participants” or “members of the public.”

### 2.3 Data analysis

With permission, all interviews were audio recorded and then transcribed by professional transcribers. Transcripts were checked for accuracy by a member of the research team, who also removed any identifying information. We used thematic analysis [47] and NVivo v12, a software program designed to facilitate analysis of qualitative data. After initial familiarisation, data were organised thematically under each policy suggestion. The transcripts were then coded line‐by‐line by one researcher using primarily deductive coding based on the policy suggestions. After this, commonalities and differences across the policies and across participants were explored, through a process of comparing coding between multiple transcripts. Findings were discussed with the research team and refined through an iterative process.

## 3 RESULTS

### 3.1 Restricting alcohol advertising and sponsorship

In accordance with existing evidence on the relationship between alcohol advertising and alcohol use, all professional stakeholders suggested alcohol advertising was a clear priority for policy change. Therefore, discussions with professional stakeholders focused on the feasibility of restricting alcohol advertising. Some suggested a ban on all alcohol advertising (“*it is easier to do a complete ban*”, S4) while others favoured the suggestion to restrict advertising in public spaces, sports events and festivals as “*a first step before you start trying to ban all alcohol advertising*” (S6). Some public stakeholders were also aware of the relationship between alcohol advertising and alcohol use, and were in favour of restricting advertising (“*A really good first place to start [as] it's what encourages people* [to drink]” Ellie, FG6). Others emphasised personal responsibility in relation to alcohol use and a belief that drinking is about *“self‐control”—“if you're gonnae* [going to] *buy alcohol, you're gonnae buy alcohol”* (Harry, FG1). These participants were sceptical about the potential for advertising restrictions to reduce drinking among adults as “*it's* [advertising] *not forcing you to go out and buy it*.” (Veronica, FG1). Although many agreed “*it's a good idea to disassociate alcohol with sporting events*” (Leo, FG9), members of the public had concerns about the effectiveness of banning advertising at sporting events and festivals as the relationship between music, sport and alcohol was perceived as complex and socially embedded:

Gregor (FG11): “I don't think that would really make a difference. If somebody's going to a festival or an event, they are probably more likely to take drink. It doesn't matter if it's advertised or not.”

Kirsten (FG4): “I do think there's a very interesting dynamic between sport and alcohol, it's … wrapped up in this cultural ‘celebrationary’ stuff, but equally it feels contradictory that sport is about enjoyment, but it's also about health … either as a spectator or a participant … trying to do something healthy that either brings you joy or brings you community, or brings you fitness.”

A few focus group participants were concerned that banning sponsorship of sporting events and festivals would result in negative outcomes:

Ellie (FG6): “If you think aboot [about] it this way, you wouldnae [wouldn't] have T in the Park^ii^ [music festival sponsored by Tennents a leading brand of beer in Scotland and a subsidiary of AB InBev] for a start. How much of Scottish football is sponsored by alcohol … And while I'm all for banning advertising, I think when it comes tae [to] sponsorship, you need to be balancing that against the benefit because we don't want to be punishing all football fans or all music fans, because Tennent's cannae [can't] put on a music festival this year.”

People living in deprived areas drew parallels between the advertising of alcohol and other unhealthy commodities, specifically tobacco and gambling. Their discussions suggested that alcohol is not an ‘ordinary commodity’ (a phrase commonly used by the professional stakeholders in our sample, and by public health actors [48]) and policies should target alcohol advertising in a similar way that tobacco and gambling advertising have been addressed in Scotland:

Josie (FG3):“I do not understand why they're allowed to advertise alcohol on TV, but not allowed to advertise cigarettes.”

Focus group 1:

Veronica: “They wouldnae [would not] let cigarette companies on the [football] t‐shirts, so … I wouldn't let alcohol on it either.”

Harry: “What's the difference between alcohol and gambling sites? They cover up the gambling sites when they go to certain age groups and certain competitions. But why do they no’ do that with alcohol, ‘cause there's an age limit on that as well.”

Although we did not ask about online alcohol advertising, both professional and public stakeholders spontaneously mentioned it as a barrier to restricting alcohol advertising. They debated the feasibility of restricting online advertising, including user‐generated content:

S4: “Regulating advertising, from an internet perspective, where exactly is the ban to be? Is it to be legislated for in Ireland, let's say, but what if that advertisement actually originates in the UK or America or whatever? You can see how difficult it is to actually regulate the internet because it's not confined to particular jurisdictions.”

Emma (FG8): “That [alcohol advertising] would be very hard to reduce, especially on social media, you see all these influencers, when they go Ibiza … They get paid to show ‘we drink, they drink’. (…) They will pay one influencer to show up with the alcohol and the sales will triple [compared to advertising] on television.”

### 3.2 Regulating availability: banning alcohol sales near schools

Discussions naturally focused on discussions around banning alcohol sales near schools, as this is where children spend a large part of their time. Generally, the professional stakeholders in our sample were supportive of *“an exclusion zone”* (S2) for alcohol sales around schools and viewed it as a “*a step in the right direction”* to reduce children's exposure to alcohol:

S1: “I think because alcohol is A) a harmful product and B) an age‐restricted product, that it makes sense to minimise the exposure that children have to alcohol adverts and sales … So, if a child is popping into their local shop for a snack after school, they shouldn't be exposed to shelves and shelves of alcohol. It should be seen as a separate product.”

Similarly, some of the public stakeholders believed the policy could reduce children's exposure to, and subsequently their views on alcohol:

Carly (FG9): “Even these small corner shops near schools where some people might go on their lunch … you're then seeing all these drinks that are targeted at younger people. I just think it's completely inappropriate because it's hard to change adults’ mindset towards alcohol, so I feel like our only real hope is changing young people's perspective on it.”

However, professional and public stakeholders questioned the potential effectiveness of such a policy, given children's exposure to alcohol in their wider environment and the relationship between alcohol availability and advertising. For example, professional stakeholders reflected on the fact that “*children don't actually just remain within local areas*” (S7) and a child may take a bus “*seven kilometres away, and then passes so many adverts on the bus*” (S13). They also questioned the feasibility of this policy in city environments: “*if you were to draw a radius around every school in the city of three hundred metres, I think you would find a vast majority of the city would be an exclusion zone*” (S14). Public stakeholders also emphasised the relatively greater importance of a child's home environment:

Chelsey (FG6): “I think that most young people get the majority of their exposure to alcohol, and their attitudes towards alcohol from home.”

There was a view among some public stakeholders that there was no need to ban the sale of alcohol near schools because it is an age‐restricted product. They also feared it would have a negative impact on local businesses:

Veronica (FG1): “It's not like the school kids can go in and drink it, or buy it. They've got two shops next to each other, one sells alcohol, one doesn't. I don't think it matters to my kids which one they go in, regardless.”

Focus group 9:

Debbie: “To be fair, I think it would put some corner shops out of business. Corner shops are looking to operate all day and they're selling the kids the sweeties during the nine ‘til three [9am‐3pm], but they also have the sales from the alcohol later in the day to be profitable.”

Leo: “As long as the alcohol's not being sold to the same children later.”

### 3.3 Regulating availability: limit the number of places that sell alcohol

Professional stakeholders viewed high alcohol outlet density as a public health issue. Limiting the number of outlets in specific neighbourhoods was considered an important policy suggestion, especially for areas of high deprivation given that unhealthy commodities cluster in such areas [49]. Public stakeholders highlighted that there are already “*too many shops selling alcohol*.” Despite this, high alcohol outlet density was rarely seen as a public health issue among public stakeholders. The dominant narrative was that reducing the number of outlets selling alcohol (off‐ and on‐premise alcohol outlets) in specific neighbourhoods would not reduce alcohol use among adults. Personal responsibility was emphasised again:

Jessica (FG10): “I think if you're gonna [going to] go for alcohol you're gonna get alcohol. It doesn't matter how many places are open … if there's one store or five stores, I don't think it'll make a difference.”

Concerns about the effectiveness of restricting alcohol availability (particularly off‐premise outlets) often relied on a narrative of “othering” where other people (usually those perceived to be dependent on alcohol) would travel further to obtain alcohol. In the excerpt from focus group 4 below, Rob drew on this narrative while Amy reflected on her own behaviour suggesting she would purchase more alcohol if availability was reduced:

Focus group 4:

Rob: “Well it depends whether you're talking about drinking or problem drinking. If someone's got a medical condition, which is alcohol dependency, that's not going to stop ‘em, they're going to go, they'll walk an hour to get it. And they'll buy as much as they need or can afford at that given moment in time. So it's a specious argument at best.”

Amy: “If I knew that there was limited availability … if we had one shop in this area that was selling it, I'm more likely to buy more in a oner [at one time] than I would if there was more availability. Which could be harmful … it could lead to something damaging … So I agree with the general thoughts so far. I'm not sure how effective that would be as a policy.”

Although public stakeholders generally did not think restricting off‐premise alcohol availability would reduce drinking among adults, some believed it might be effective in changing young people's attitudes to alcohol use:

Tim (FG2): “We don't want an abundance o’ places where you can buy alcohol, because that's where you start getting … young people, somebody will get them it … and if you look older than you are, it's easier to get it (…) so you're hoping that young people are no’ exposed to it as much, so they're no’ really bothered about it … and maybe in the likes o’ ten years, the level [of alcohol use], it'll be a way down. ‘Cause it's not an overnight fix, is it? You've got to go through a kinda [kind of] a generation thing tae [to] get the levels doon. [down]”

Those in favour of limiting the number of outlets in specific locations believed that a large number of pubs and clubs created a “*more dangerous environment for a neighbourhood*” (Emma, FG8). Therefore, limiting on‐premise outlet density was perceived to have potential for reducing anti‐social behaviour and increasing perceptions of safety:

John (FG3): “I would seek to limit pubs but maybe not so much off‐licences. Because generally speaking, the amount of antisocial behaviour increases with the amount of pubs but not necessarily off‐licences.”

Female participants in particular expressed concerns about safety in areas of high on‐premise availability. They had conflicting views about the policy, suggesting that although limiting the number of on‐premise outlets may increase of safety among people walking on the streets, it may make the environment inside premises less safe:

Focus group 9:

Carly: “Young people work in town and they'll be heading home sometimes at the same time people are drunk in town. A young woman myself, I would not feel safe walking through town with the amount of drunk men.”

Researcher: “So do you think having fewer pubs or bars would increase that feeling of safety?”

Carly: “Maybe, in the city centre, it might. But then you might also have the opposite effect of too many people in the one area, and then people are all bumping into each other, getting annoyed, getting aggressive. You might not be safe when you're inside.”

One participant in the focus groups emphasised creating healthy environments, rather than focusing on restricting availability of a single commodity:

Kirsten (FG4): “I think that there certainly should be attention to how much alcohol is available in a particular area, and why. And I think this, however, needs to line up with what we also know about food and nutrition … we know that in areas of deprivation there's much more fast food, and less walkable distances to affordable supermarkets and fresh food. So I think, as part of a more general preventative health knowledge, being aware of the choices that you're making, I would be more inclined to be more supportive of this suggestion than some of my colleagues here tonight.”

Both groups of participants (professional and public stakeholders) reflected on whether reducing the number of outlets in specific neighbourhoods was simplistic. For example, one professional stakeholder suggested that supermarkets can provide benefits for communities, particularly deprived communities (e.g., fresh products, job creation) and this needed to be balanced against potential alcohol‐related harms:

S14: “If you have what appears to be a blanket ban on overprovision localities and there's a presumption against the grant of further off‐sales licences in these areas of deprivation, is that potentially sending out a message that you don't want your [supermarkets] to come in, and those communities then possibly lose out? They don't get the nice new modern convenience stores, and they are stuck with some really old, quite tatty shops, because no new licences are getting granted. There are potential downsides to this as well.”

Some public stakeholders believed that restricting alcohol availability is “*taking away your freedom o’ choice*.” They drew on the narrative of “othering” suggesting that this policy would target a minority of the population who experience alcohol‐related harm, while “restricting choice” for other people:

Rob (FG4): “And it's treating the people that have alcohol dependency, and make it difficult for them to buy. Quite how you do that's another story, but restricting the availability of a legal product to the whole population because there's a minor amount of people with a problem drinking habit, that's a step too far for me.”

Online alcohol availability was mentioned spontaneously by both professional and public stakeholders. Online sales were perceived to undermine attempts to reduce the physical availability of alcohol:

S13: “We've got an internet savvy population now … they can just get a huge amount of alcohol. And as long as they're an adult, it'll be delivered to their home.”

Natalie (FG1): “Even all your big supermarkets for your online shops, you can get it [alcohol]. So I don't know if that [limiting the number of outlets] would overall make a big difference.”

### 3.4 Regulating availability: physical separation of alcohol from other products

Professional stakeholders criticised how alcohol was seen as an “ordinary” or “everyday commodity.” Alcoholic beverages of any strength are available to buy as part of a regular shop in Scotland (compared to being separated either behind a physical barrier or for sale in alcohol‐only outlets), and this was believed to reinforce this perception:

S4: “Seeing alcohol for sale in ordinary shops alongside milk, it's telling the next generation that it is an ordinary product”

Therefore, professional stakeholders favoured the physical separation of alcohol from other products as a way to de‐normalise alcohol as an everyday commodity (e.g., “*outta sight, outta mind*”, S2). They also suggested that this policy could protect children and people with alcohol dependency by reducing their exposure to alcohol:

S6: “I think it's a good policy … from two angles … by reducing the normalisation of alcohol and children just seeing it all the time. And also for people in recovery … if you go into a supermarket, you can avoid seeing the alcohol. If you go into a small shop you can't, ‘cause it's quite often right next to the till.”

In contrast, the dominant narrative among public stakeholders was that selling alcohol behind a physical barrier would not reduce drinking among adults. The topic of physical separation generated most discussion in our focus groups. Although participants did not appear to change their view on the policy during the course of the focus groups, they felt comfortable challenging each other's opinions. Public stakeholders emphasised personal agency in relation to purchasing alcohol:

Zara (FG7): “I don't think that would change much, to be honest. If you're of age and drinking, you tend to know what you like to drink, so you're just gonna ask for it anyway.” (“Mm‐hmm” indicating agreement from the group).

However, some public stakeholders believed that reducing exposure to alcohol could protect children:

Carly (FG9): “I don't think it would be very effective on people that regularly drink, but I think it could be effective on younger people …before age thirteen, fourteen. (…) if you grow up and these things are sort of restricted and you just generally don't see them, it makes you not care about it as much but also it makes it seem more serious, like it's more of a big deal, which I think is important.”

Tim (FG2): “Oh aye, when something's no’ visual to you, you're just gonnae walk right by it … ‘Cause some of the alcohol, it looks like a bottle o’ pop [sweet, fizzy soft drink] and I daresay that is a ploy o’ whoever's making it … to draw young people in. I definitely think it should be hid[den], you know? Which would be a lot better.”

Although participants generally agreed that the policy would not reduce drinking among adults, there were discussions about its potential to protect children, demonstrating that when viewed through the paradigm of child protection, restrictive policies were often more palatable. This is illustrated in the excerpt below from focus group 6. Ryan perceived that this policy would penalise the majority of people for the behaviour of a minority. In contrast, Ellie suggested the purpose of the policy was to reduce children's exposure to alcohol and challenge perceptions that alcohol is an ordinary commodity (in line with professional stakeholders’ views):

Focus group 6:

Ryan: “We're gonnae get to a stage in life that … what do we cover up? … food that's no’ healthy for you? I just feel that we're getting intae a situation where it's a very small minority o’ kids that are maybe out o’ order or unsociable and that's affecting the whole o’ society.”

Ellie: “What we are talking about … is challenging the normality, and the fact that alcohol seems to be a core part of what we do. Moving alcohol to the back o’ a shop so that you can avoid it, if you're not specifically there for it, I don't see how that would affect anybody…if it's at the front of the shop and it's got all this colourful bright packaging, it's almost like advertising. And I don't see the need to have that sort of advertising in an area where a large proportion of the people that are gonnae see it aren't actually old enough to use it responsibly if you're not specifically there for alcohol … you shouldn't have to walk past it wi’ your kids.”

Others rejected the argument about protecting children, and believed hiding alcohol can make it look *“desirable”* as *“it gives it a grater allure”* and *“an air of mystery around it”*:

Lewis (FG8): “When I was younger, I knew it was illegal. I started going into pubs because I was quite tall when I was fifteen, and playing pool and getting served (laughs). I just don't think that hiding it from children, when actually alcohol's a reality of life, that that's gonna work at all. It could even have the opposite effect. I mean, at that age … quite a lot of us are exploring ourselves.”

One participant suggested it can stigmatise people:

Lewis (FG8): “To me I think it's a bit shaming … It's saying, “This stuff's illegal. It shouldn't be seen.” And I don't think shaming is the way forward for any kinda public policy in terms o’ alcohol.”

## 4 DISCUSSION

This is the first study to explore consensus and divergence in the views of public and professional stakeholders on two key alcohol control policies: advertising and availability. There was general consensus that regulation of alcohol advertising is an important policy priority, although there was some concern among public stakeholders about the feasibility of implementing advertising interventions and the potential unintended consequences of disrupting sponsorship revenues. While professional stakeholders were in favour of regulating alcohol availability, public stakeholders had misgivings about potential policy feasibility and effectiveness. Despite these concerns, when prompted to discuss specific interventions, similar views about the importance of protecting children and achieving cultural change in attitudes towards alcohol emerged.

All professional stakeholders readily agreed that regulating alcohol advertising is a clear priority for policy change, given strong evidence on the relationship between alcohol advertising and alcohol use [50]. While professional stakeholders focused on the best way to restrict advertising (through a ban or separate control measures), public stakeholders discussed the cultural norms contributing to perceived normality of alcohol use in Scotland, especially in relation to sport and music. There was an acceptance that encounters with alcohol advertising and sponsorship at sport and music events are integral to the overall event experience. This is in line with existing research highlighting the social and cultural significance of alcohol consumption within football (e.g., in the UK: [51]; in Sweden: [52]) and music (e.g., in the UK: [53, 54]; in Australia: [55]). For example, a study of UK music festival attendees found that people associate the word ‘fun’ with certain alcohol brands, suggesting sponsors have been successful in linking brands with consumers’ experience of entertainment [56]. Although most public stakeholders appeared supportive of policies to regulate alcohol advertising, they were less supportive of potential bans on alcohol sponsorship, and there was a perception that sponsorship was necessary to ensure the viability of music festivals.

Professional stakeholders viewed alcohol availability as a public health issue. They perceived alcohol as a discrete product, different from food and other drinks, and suggested that interventions need to challenge the perception of alcohol as an ‘ordinary commodity’. This is in line with existing scientific knowledge and public health framings of alcohol as a determinant of ill‐health and a contributor to health inequalities [57, 58]. Therefore, professional stakeholders were supportive of policies to reduce alcohol outlet density and introduce a physical separation for alcohol products in shops. Public stakeholders viewed the suggested availability interventions as relatively simple attempts to influence a complex social issue. Previous research has also found that policies restricting alcohol availability are less favoured among the public [17, 26, 59]. Our focus group participants perceived that societal alcohol problems result from behaviours of a minority of the population (see also [26]). When discussing the different availability policies, public stakeholders tended to focus on ‘others’, particularly harmful and dependent drinkers, while rejecting policies that might impact on their own drinking. The concept of ‘othering’ refers to identifying others as different to one's self [60]. This perspective is similar to alcohol industry framings of alcohol problems, which suggest that alcohol‐related problems are restricted to a minority of drinkers and highlight the positive effects of alcohol on society, the economy and those who drink moderately [14]. This view contradicts scientific evidence that no level of alcohol consumption is safe [58]. Public stakeholders were less supportive of policies to reduce alcohol availability and visibility, as these were seen to impact on the freedom of choice of the majority of the population, while being perceived as ‘ineffective’ for ‘others’ who would obtain alcohol despite availability restrictions (see also [26] for similar narratives among adult drinkers in England and Scotland).

Public health stakeholders in our study also discussed trade‐offs between potential health gains (i.e., reducing alcohol use and harms) and losses, especially freedom of choice and economic losses for small retailers. This has implications for alcohol control policy, as competitive framings of the same issue can undermine efforts to gain public support [61]. For example, previous research shows people lack in‐depth understanding of minimum price policies and rely on their personal beliefs [62]. It is important for scientific evidence to be disseminated in a comprehensive way that includes both the benefits and potential risks of any policy, while considering people's concerns about potential negative or unintended effects of alcohol policies. Storvoll et al. [63] suggest that strengthening the public's beliefs in policy effectiveness could increase support for restrictive alcohol policies. It is also worth noting that public opposition towards a policy can decline once the policy has been introduced, as was the case with minimum unit pricing and smoke‐free legislation in Scotland [64, 65], and alcohol warning labels internationally [66]. This can encourage governments to consider the implementation of alcohol interventions with low public support, because support may increase following implementation.

Previous research suggests that interventions targeting children and young people are more strongly supported by the general public [59]. Although our focus groups participants were sceptical that the proposed availability and advertising interventions could reduce drinking among adults, they showed support for policies focused on children and young people. A recent Scottish Government consultation on alcohol advertising shows that children who were part of the consultation, reported awareness of the role alcohol plays in the cultural life of Scotland and the harms caused by alcohol [67]. They were supportive of policies to restrict alcohol sponsorship in sport, restrictions on advertising in places frequented by children, and restrictions of online advertising of alcohol. This highlights the importance of including children and young people's voices in the policy making process.

Although public and professional stakeholders were in favour of policies to protect children, they expressed less support for a specific intervention to ban the sale of alcohol near schools. They reported different reasons for this, including children's exposure to alcohol in their environment (e.g., home, outside school) and the importance of alcohol sales for the economic viability of smaller shops. Professional stakeholders questioned the potential effectiveness of this intervention highlighting the relationship between alcohol availability and advertising, and children being exposed to advertising on their way to school. They also suggested that the policy would not be feasible in an urban environment where there is high outlet density. Existing research shows that children are often exposed to alcohol and alcohol‐related products both inside and outside the home, including built and media environments [68, 69, 70]. Children living in lower socioeconomic areas are particularly vulnerable to alcohol exposure in their built environment [71]. Evidence from longitudinal studies consistently shows that exposure to alcohol advertising is associated with a greater likelihood of adolescents consuming alcohol, and with heavier drinking amongst adolescent baseline drinkers [72].

It is well established in Scotland that there are disproportionately more alcohol outlets in disadvantaged than affluent neighbourhoods [43, 49], and both professional and public stakeholders discussed this. Both groups also highlighted the importance of healthy environments, with restrictions on all unhealthy commodities (i.e., alcohol, tobacco, gambling), while acknowledging the role of the online environment. Digital advertising has extensive reach and allows marketers to interact with users and build brand identities [73, 74]. One topic that has received little attention, and was mentioned by public stakeholders in our sample, is the role of influencers in advertising alcohol across a variety of social media platforms. There is a need for more research to understand online advertising and availability, especially given online alcohol purchasing has been increasing internationally [75]. We have previously reported that public health stakeholders have expressed concerns about whether restricting physical availability of alcohol may lead to an increase in online availability [38]. From a policy perspective, it might be challenging to address online availability and more research is needed to inform future policy discussions. Colbert et al. [76] recently suggested the introduction of mandatory responsible service of alcohol training for all alcohol home delivery drivers as a potential way to mitigate the risks posed by online alcohol delivery services. Fitzgerald et al. [77] propose a ‘sweetspot’ intervention, which could allow on‐trade premises to offer take‐away alcohol while regulating speed/time of delivery, age verification, staff training and marketing of alcohol sold remotely.

### 4.1 Study limitations

This study provides insight into public opinions about restricting alcohol advertising and availability. We made a decision to conduct the professional stakeholder interviews first, followed by the public focus groups. The material from the stakeholder interviews inevitably shaped our direction in the focus groups. Although this is similar to existing research [78], it may be a limitation and in future research it would be interesting to consider public views first and then seek to explore how professional stakeholders would respond to these to assess consensus and divergence of opinion. The use of focus groups allowed discussion of alcohol interventions from various perspectives with participants challenging each other's arguments. We did not observe participants’ opinions changing during the course of each focus group. While we collected data from diverse populations in terms of personal characteristics (e.g., age, alcohol use) and neighbourhoods (e.g., rurality, deprivation, retail landscape), we do not know to what extent the experience of Scotland may be transferable to other international contexts. Research in countries with different alcohol cultures and legislation would further contribute to understanding public support for alcohol control interventions. Our sample did not include participants with ‘possible dependence’ on alcohol (as per Alcohol Use Disorders Identification Test), so does not reflect the perceptions of this section of the population. We were able to recruit public health stakeholders, some of whom had been involved in alcohol advocacy, but unable to recruit alcohol retailers who may well have offered different views on alcohol control [79].

## 5 CONCLUSION

This is the first study to explore consensus and divergence in the views of professional and public stakeholders about restricting alcohol advertising and availability. The findings suggest that the general public has nuanced understanding of policy feasibility and effectiveness. While professional stakeholders framed the proposed interventions as important first steps to protecting children and reducing alcohol use, the public stakeholders had more reservations. Scepticism about policy feasibility and potential effectiveness is likely to be a barrier to public support for policies to reduce alcohol availability. However, policies framed as protecting children were likely to receive more public support. It is important to improve communication of public health evidence by considering people's concerns about the effectiveness and potential negative consequences of alcohol control policies while considering the wider social and economic context of alcohol in people's lives.

## AUTHOR CONTRIBUTIONS

Elena D. Dimova: Data curation; investigation; formal analysis; resources; software; validation; visualisation; writing—original draft. Niamh K. Shortt: Conceptualisation; funding acquisition; methodology; project administration; supervision; resources; formal analysis; validation; writing—review and editing. Matt Smith: Data curation; investigation; writing—review and editing. Richard J. Mitchell: Conceptualisation; funding acquisition; formal analysis; validation; writing—review and editing. Peter Lekkas: Formal analysis; validation; writing—review and editing. Jamie R. Pearce: Conceptualisation; funding acquisition; formal analysis; validation; writing—review and editing. Tom L. Clemens: Conceptualisation; funding acquisition; formal analysis; validation; writing—review and editing. Carol Emslie: Conceptualisation; funding acquisition; methodology; data curation; formal analysis; project administration; supervision; validation; writing—review and editing.

## FUNDING INFORMATION

The study was funded by Economic and Social Research Council, Grant/Award Number: ES/S016775/1. RM is funded by the UK Medical Research Council (MRC) Places and Health Programme (MC_UU_00022/4) and the Chief Scientist Office (CSO) (SPHSU19) at the MRC/CSO Social and Public Health Sciences Unit, University of Glasgow.

## CONFLICT OF INTEREST STATEMENT

The authors declare no conflict of interest.

## Supporting information


**Data S1.** Supporting information.

## Data Availability

The data that support the findings of this study are openly available on ReShare at https://reshare.ukdataservice.ac.uk/856665/#:~:text=Understanding%20the%20Lived%20Experience%20of%20Alcohol%20and%20Tobacco.

## Endnotes

^i^
Datazones are small areal‐units used for routine reporting of local data. They typically contain 500–1000 residents and are designed to distinguish demographically, socially and geographically distinct ‘neighbourhoods’.

^ii^
T in the Park was replaced by TRNSMT in 2017, whose sponsors include Bacardi and Jägermeister.
