# Supplementary figures and images for: Public and professional stakeholders’ perceptions of alcohol advertising and availability policies: A qualitative study
Source: Drug Alcohol Rev. 2024 Oct 28;44(1):104–18. doi: 10.1111/dar.13972 (PMC11743051; doi:10.1111/dar.13972)

Supplementary material 1. Infographics for alcohol interventions


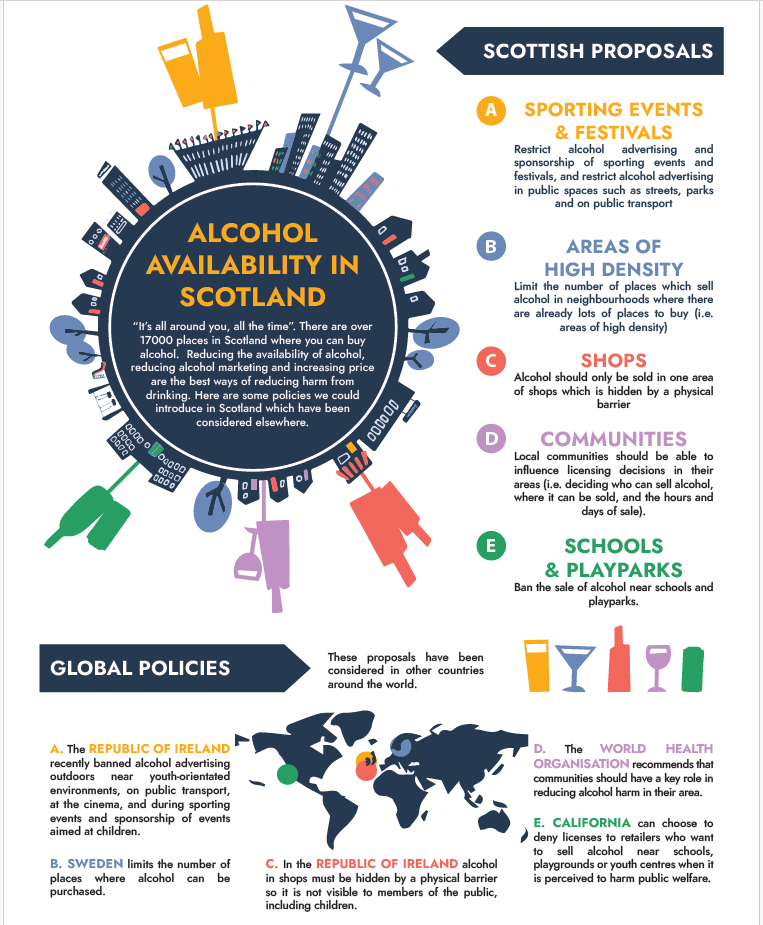

Supplement: Supplementary file 1 — Data S1. Supporting information. [file DAR-44-104-s001.docx]
